# Supplementary material for: Humoral and Cellular Immune Responses to SARS-CoV-2 in Participants with Head and Neck Cancer
Source: Viruses. 2025 Jun 13;17(6):848. doi: 10.3390/v17060848 (PMC12197323; doi:10.3390/v17060848)
Supplement: Supplementary file 1 [file viruses-17-00848-s001.zip › Marutescu et al Supple Figures 15 20250428.pptx]

## Slide 1
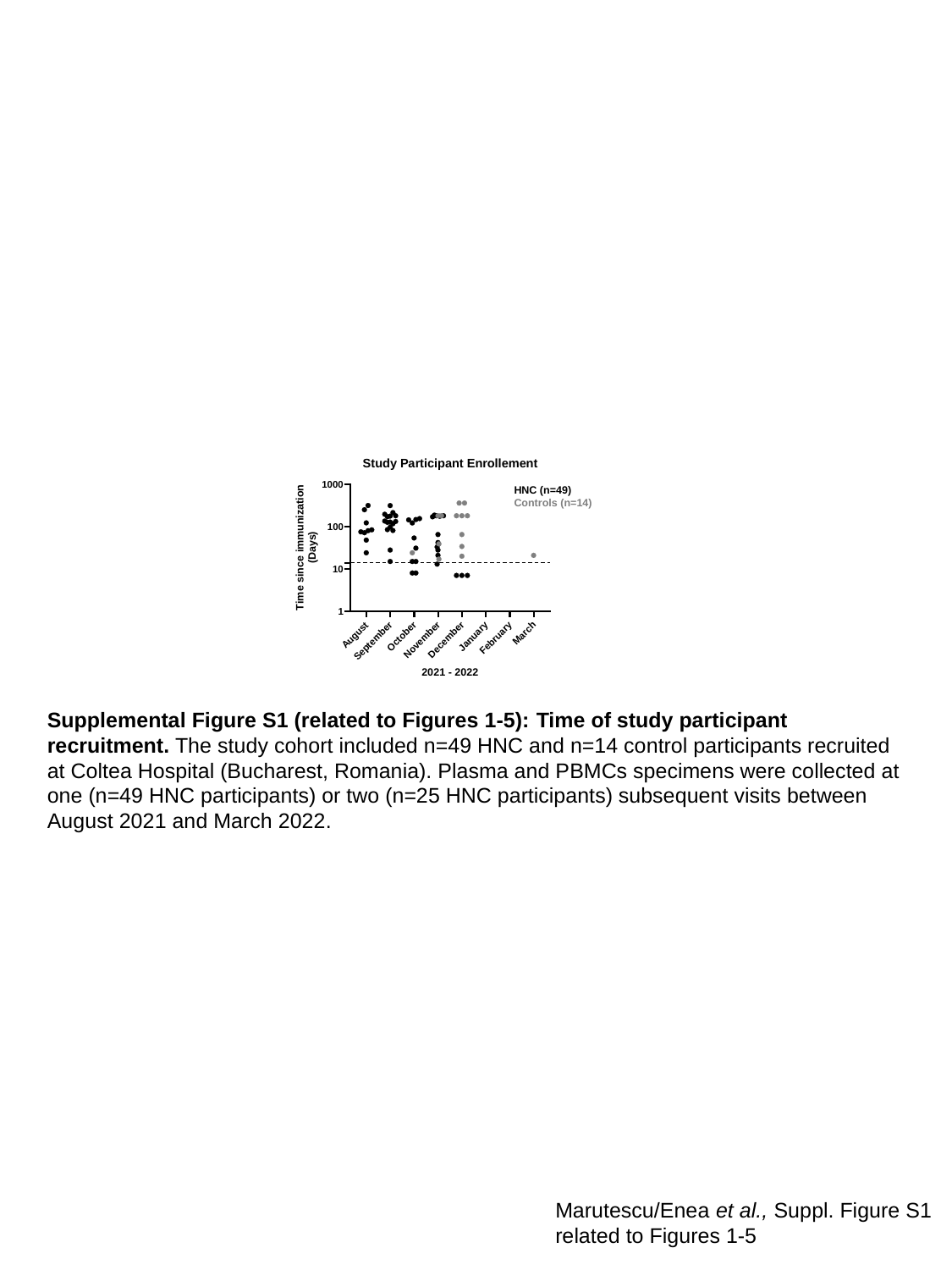

Supplemental Figure S1 (related to Figures 1-5): Time of study participant recruitment. The study cohort included n=49 HNC and n=14 control participants recruited at Coltea Hospital (Bucharest, Romania). Plasma and PBMCs specimens were collected at one (n=49 HNC participants) or two (n=25 HNC participants) subsequent visits between August 2021 and March 2022.
Marutescu/Enea et al., Suppl. Figure S1
related to Figures 1-5

## Slide 2
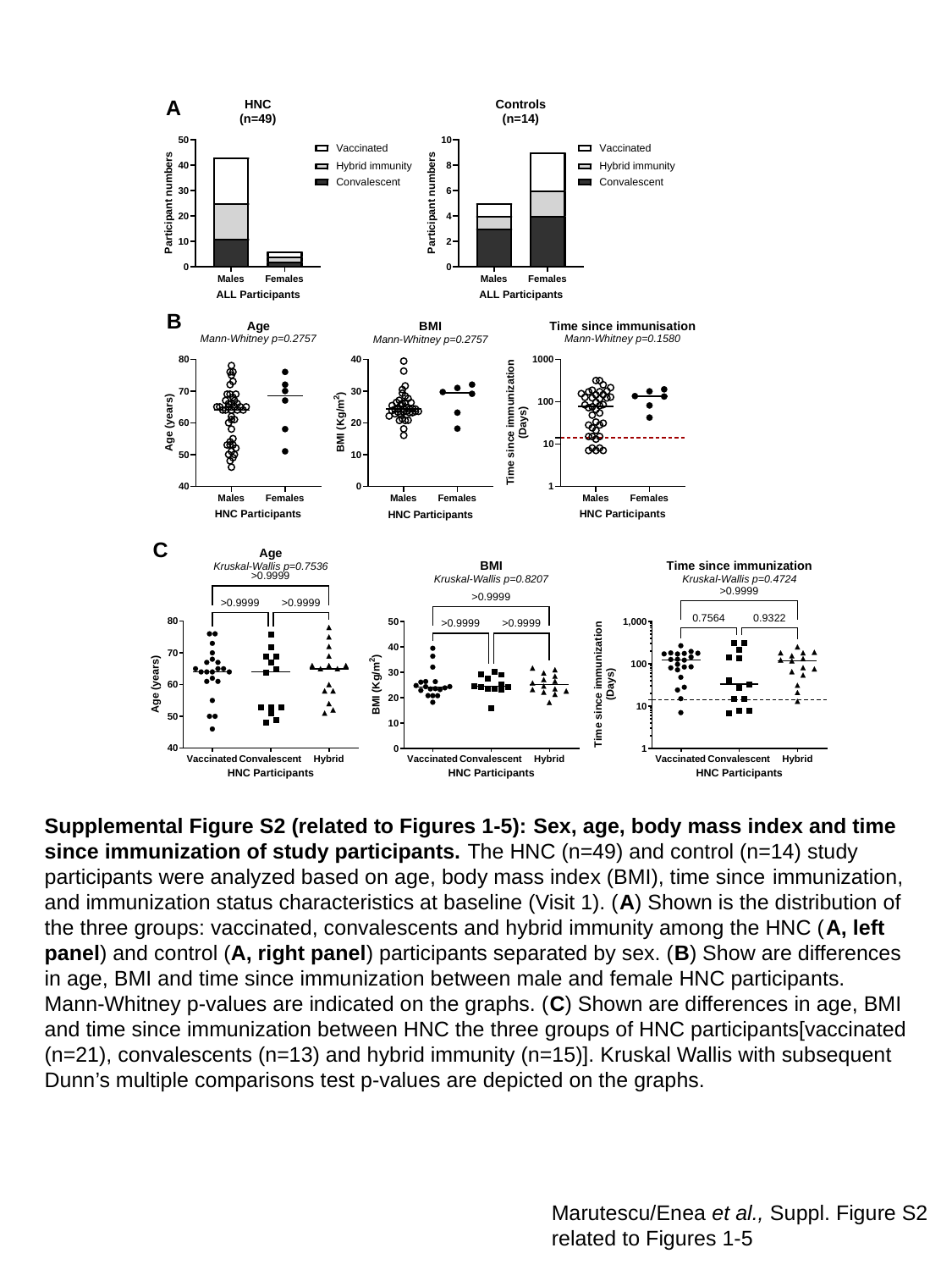

A
B
C
Supplemental Figure S2 (related to Figures 1-5): Sex, age, body mass index and time since immunization of study participants. The HNC (n=49) and control (n=14) study participants were analyzed based on age, body mass index (BMI), time since immunization, and immunization status characteristics at baseline (Visit 1). (A) Shown is the distribution of the three groups: vaccinated, convalescents and hybrid immunity among the HNC (A, left panel) and control (A, right panel) participants separated by sex. (B) Show are differences in age, BMI and time since immunization between male and female HNC participants. Mann-Whitney p-values are indicated on the graphs. (C) Shown are differences in age, BMI and time since immunization between HNC the three groups of HNC participants[vaccinated (n=21), convalescents (n=13) and hybrid immunity (n=15)]. Kruskal Wallis with subsequent Dunn’s multiple comparisons test p-values are depicted on the graphs.
Marutescu/Enea et al., Suppl. Figure S2
related to Figures 1-5

## Slide 3
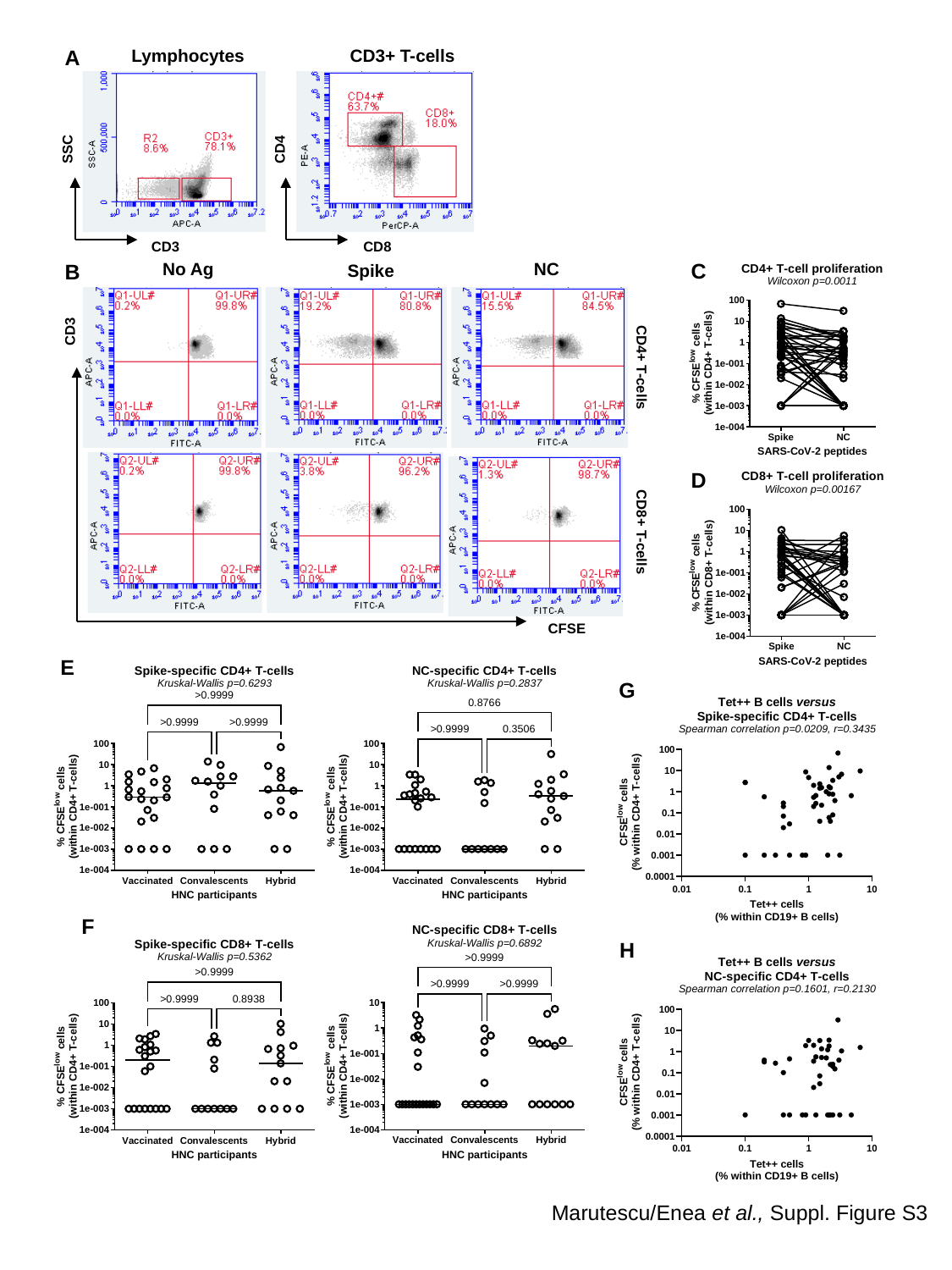

A
Lymphocytes
CD3+ T-cells
SSC
CD4
CD3
CD8
NC
C
No Ag
B
Spike
CD3
CD4+ T-cells
D
CD8+ T-cells
CFSE
E
G
F
H
Marutescu/Enea et al., Suppl. Figure S3

## Slide 4
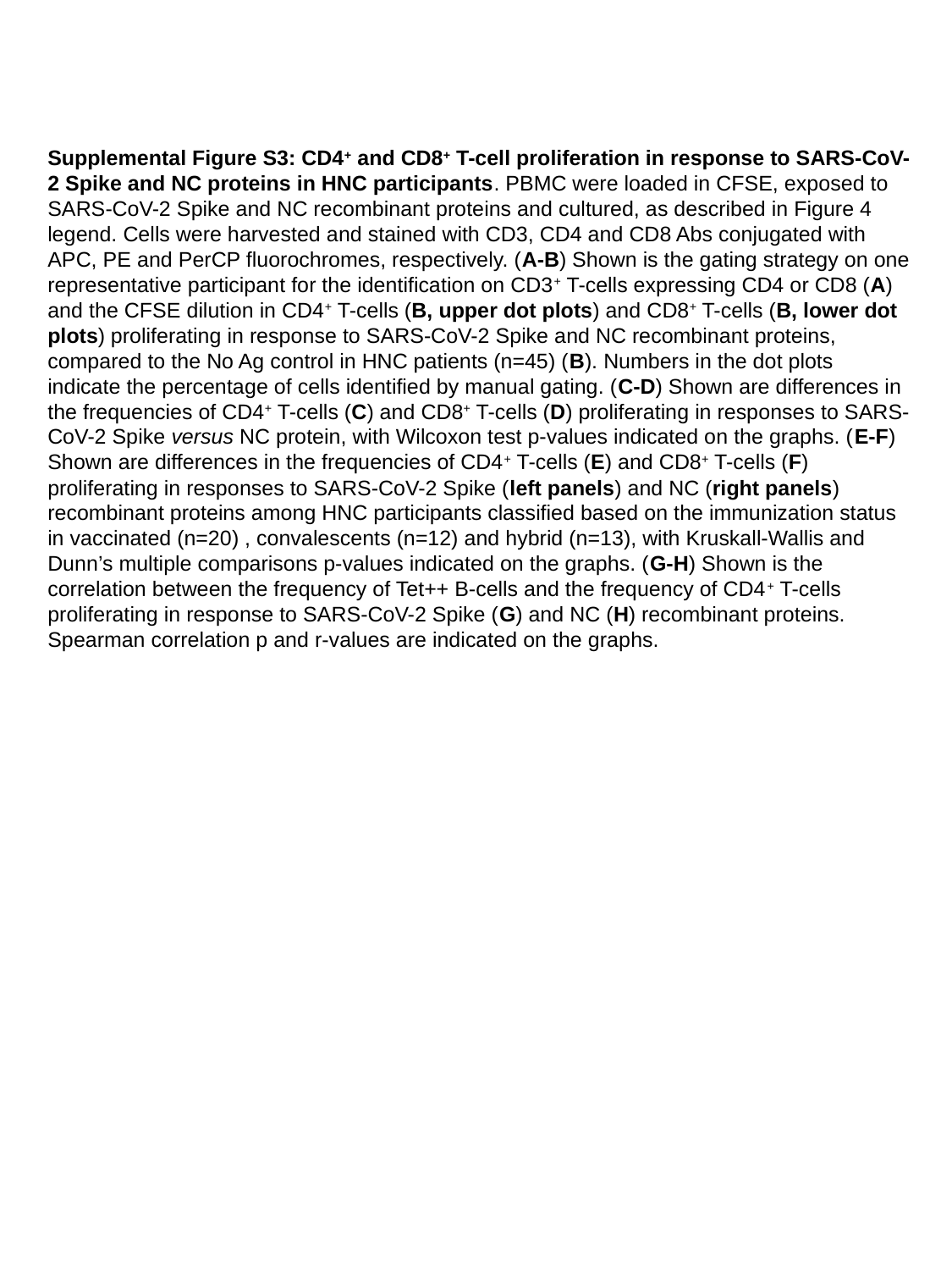

Supplemental Figure S3: CD4+ and CD8+ T-cell proliferation in response to SARS-CoV-2 Spike and NC proteins in HNC participants. PBMC were loaded in CFSE, exposed to SARS-CoV-2 Spike and NC recombinant proteins and cultured, as described in Figure 4 legend. Cells were harvested and stained with CD3, CD4 and CD8 Abs conjugated with APC, PE and PerCP fluorochromes, respectively. (A-B) Shown is the gating strategy on one representative participant for the identification on CD3+ T-cells expressing CD4 or CD8 (A) and the CFSE dilution in CD4+ T-cells (B, upper dot plots) and CD8+ T-cells (B, lower dot plots) proliferating in response to SARS-CoV-2 Spike and NC recombinant proteins, compared to the No Ag control in HNC patients (n=45) (B). Numbers in the dot plots indicate the percentage of cells identified by manual gating. (C-D) Shown are differences in the frequencies of CD4+ T-cells (C) and CD8+ T-cells (D) proliferating in responses to SARS-CoV-2 Spike versus NC protein, with Wilcoxon test p-values indicated on the graphs. (E-F) Shown are differences in the frequencies of CD4+ T-cells (E) and CD8+ T-cells (F) proliferating in responses to SARS-CoV-2 Spike (left panels) and NC (right panels) recombinant proteins among HNC participants classified based on the immunization status in vaccinated (n=20) , convalescents (n=12) and hybrid (n=13), with Kruskall-Wallis and Dunn’s multiple comparisons p-values indicated on the graphs. (G-H) Shown is the correlation between the frequency of Tet++ B-cells and the frequency of CD4+ T-cells proliferating in response to SARS-CoV-2 Spike (G) and NC (H) recombinant proteins. Spearman correlation p and r-values are indicated on the graphs.

## Slide 5
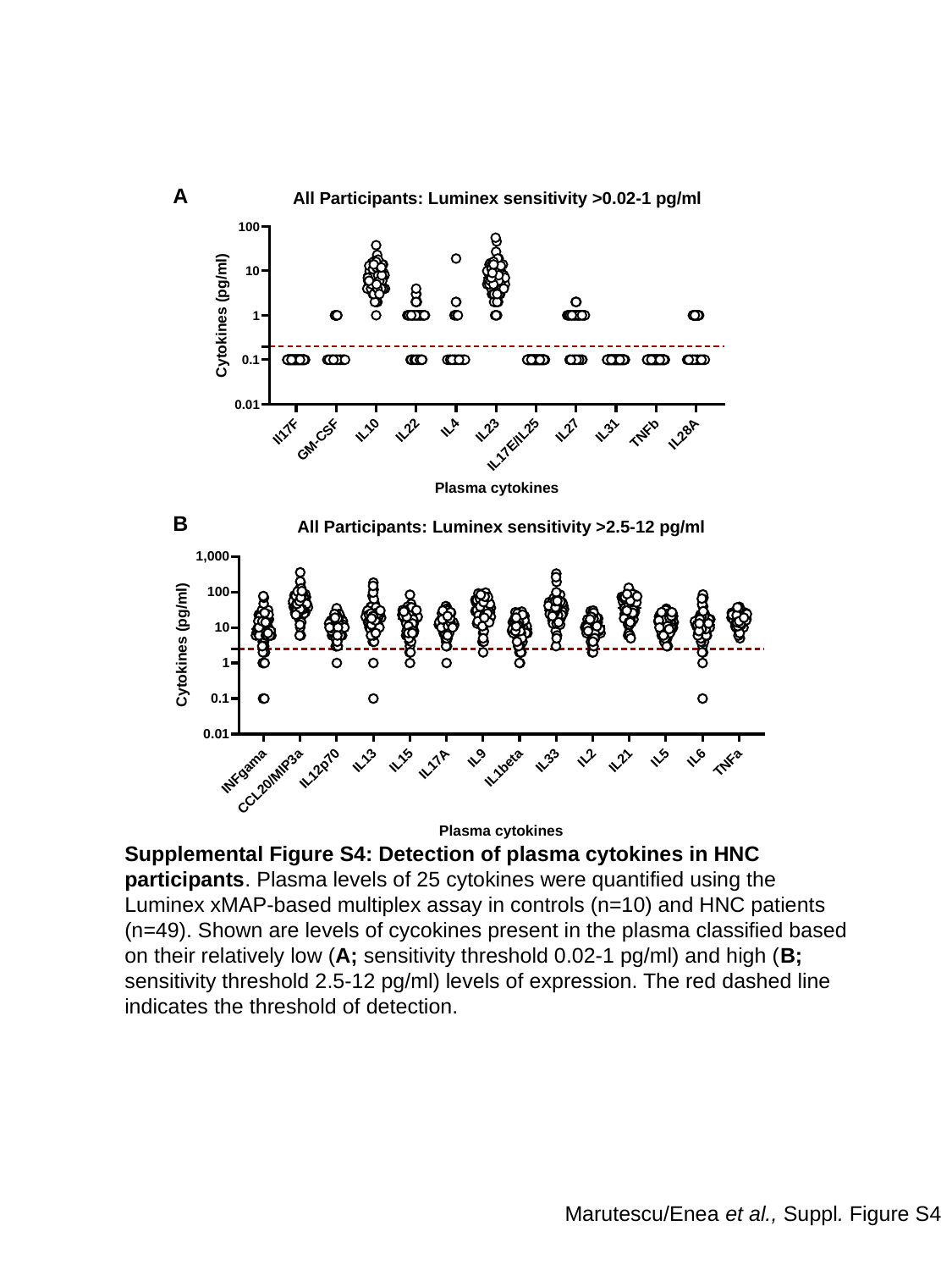

A
B
Supplemental Figure S4: Detection of plasma cytokines in HNC participants. Plasma levels of 25 cytokines were quantified using the Luminex xMAP-based multiplex assay in controls (n=10) and HNC patients (n=49). Shown are levels of cycokines present in the plasma classified based on their relatively low (A; sensitivity threshold 0.02-1 pg/ml) and high (B; sensitivity threshold 2.5-12 pg/ml) levels of expression. The red dashed line indicates the threshold of detection.
Marutescu/Enea et al., Suppl. Figure S4

## Slide 6
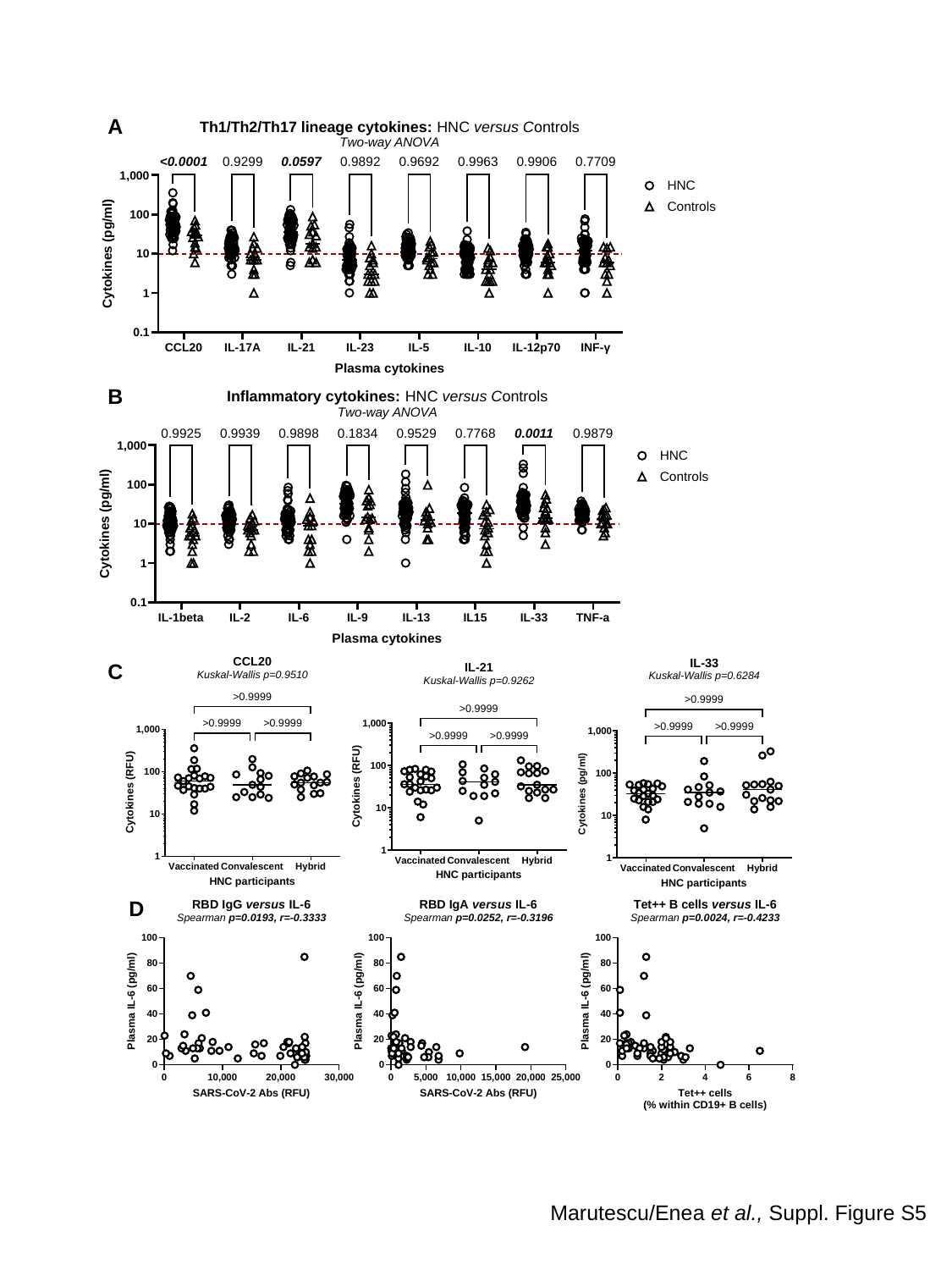

A
B
C
D
Marutescu/Enea et al., Suppl. Figure S5

## Slide 7
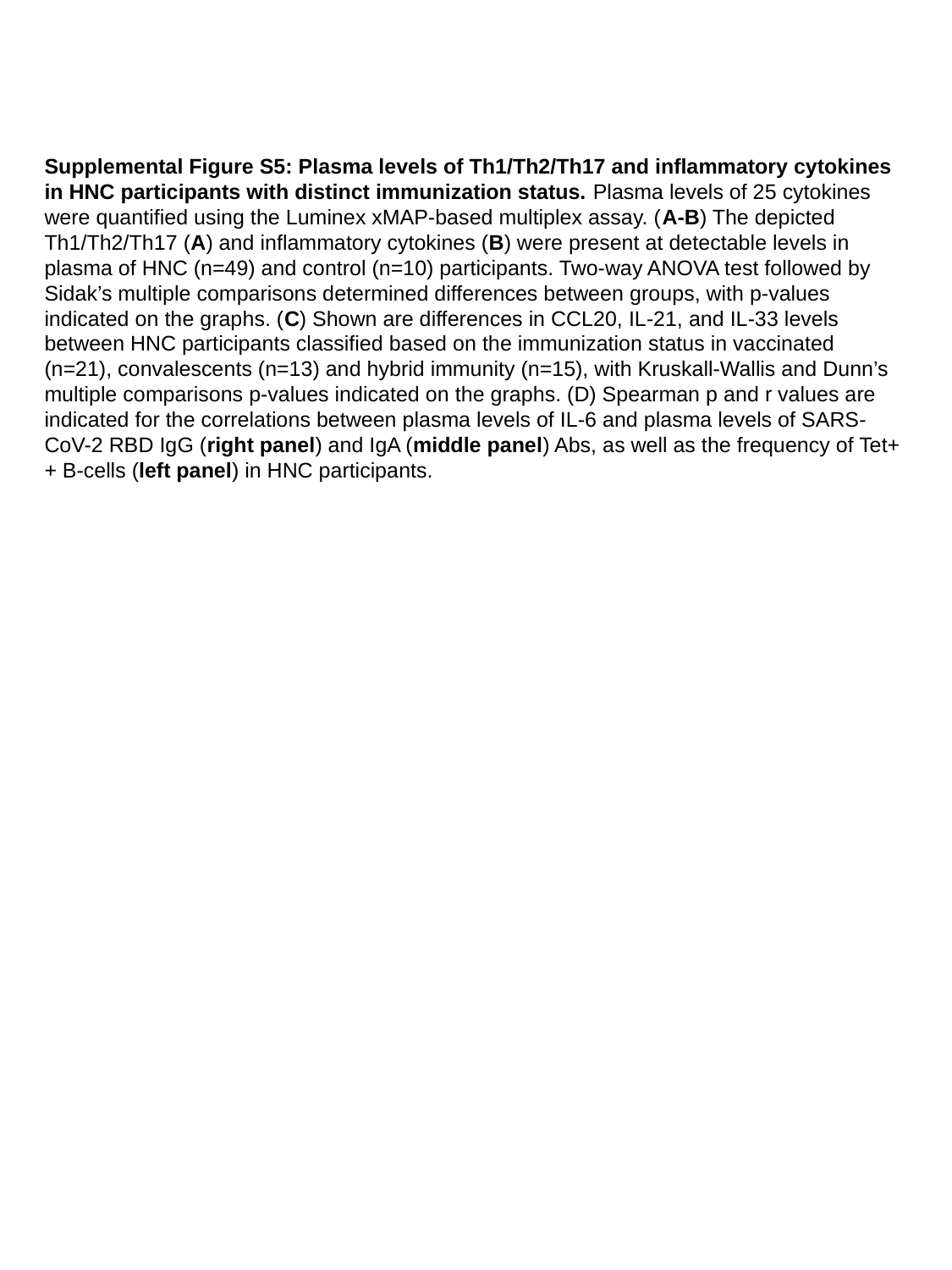

Supplemental Figure S5: Plasma levels of Th1/Th2/Th17 and inflammatory cytokines in HNC participants with distinct immunization status. Plasma levels of 25 cytokines were quantified using the Luminex xMAP-based multiplex assay. (A-B) The depicted Th1/Th2/Th17 (A) and inflammatory cytokines (B) were present at detectable levels in plasma of HNC (n=49) and control (n=10) participants. Two-way ANOVA test followed by Sidak’s multiple comparisons determined differences between groups, with p-values indicated on the graphs. (C) Shown are differences in CCL20, IL-21, and IL-33 levels between HNC participants classified based on the immunization status in vaccinated (n=21), convalescents (n=13) and hybrid immunity (n=15), with Kruskall-Wallis and Dunn’s multiple comparisons p-values indicated on the graphs. (D) Spearman p and r values are indicated for the correlations between plasma levels of IL-6 and plasma levels of SARS-CoV-2 RBD IgG (right panel) and IgA (middle panel) Abs, as well as the frequency of Tet++ B-cells (left panel) in HNC participants.
